# Intra-Genomic Variation in the Ribosomal Repeats of Nematodes

**DOI:** 10.1371/journal.pone.0078230

**Published:** 2013-10-11

**Authors:** Holly M. Bik, David Fournier, Way Sung, R. Daniel Bergeron, W. Kelley Thomas

**Affiliations:** 1 Union Council Davis Genome Center, University of California Davis, Davis, California, United States of America; 2 Hubbard Center for Genome Studies, University of New Hampshire, Durham, New Hampshire, United States of America; 3 Department of Computer Science, University of New Hampshire, Durham, New Hampshire, United States of America; 4 Department of Biology, Indiana University, Bloomington, Indiana, United States of America; University of Lausanne, Switzerland

## Abstract

Ribosomal loci represent a major tool for investigating environmental diversity and community structure via high-throughput marker gene studies of eukaryotes (e.g. 18S rRNA). Since the estimation of species’ abundance is a major goal of environmental studies (by counting numbers of sequences), understanding the patterns of rRNA copy number across species will be critical for informing such high-throughput approaches. Such knowledge is critical, given that ribosomal RNA genes exist within multi-copy repeated arrays in a genome. Here we measured the repeat copy number for six nematode species by mapping the sequences from whole genome shotgun libraries against reference sequences for their rRNA repeat. This revealed a 6-fold variation in repeat copy number amongst taxa investigated, with levels of intragenomic variation ranging from 56 to 323 copies of the rRNA array. By applying the same approach to four *C. elegans* mutation accumulation lines propagated by repeated bottlenecking for an average of ~400 generations, we find on average a 2-fold increase in repeat copy number (rate of increase in rRNA estimated at 0.0285-0.3414 copies per generation), suggesting that rRNA repeat copy number is subject to selection. Within each *Caenorhabditis* species, the majority of intragenomic variation found across the rRNA repeat was observed within gene regions (18S, 28S, 5.8S), suggesting that such intragenomic variation is not a product of selection for rRNA coding function. We find that the dramatic variation in repeat copy number among these six nematode genomes would limit the use of rRNA in estimates of organismal abundance. In addition, the unique pattern of variation within a single genome was uncorrelated with patterns of divergence between species, reflecting a strong signature of natural selection for rRNA function. A better understanding of the factors that control or affect copy number in these arrays, as well as their rates and patterns of evolution, will be critical for informing estimates of global biodiversity.

## Introduction

The ribosome is a fundamental component of the eukaryotic cell, and thus, nuclear genes encoding the ribosomal subunits have long been the focus of intensive empirical study. Ribosomal RNA genes (rRNA) are organized within the nuclear genome as tandem repeat arrays, with each repeat containing one copy of conserved coding regions (28S, 18S, and 5.8S subunit genes) and rapidly evolving noncoding regions encompassing the internal and external transcribed spacers (ITS and ETS, respectively) and intergenic spacers (IGS) [1]. The process of concerted evolution acts as a homogenizing force across rRNA repeats within a genome (thus conferring high sequence identity between arrays within a species [2]), whereas this same process effectively allows divergence in rRNA between reproductively isolated species. 

Ribosomal RNA genes have been used as markers for phylogeny reconstruction [3], diversity analysis [4,5], and genome evolution studies [6]. These loci are amenable to PCR-based assays due to their pseudo-orthology and the large amount of existing data readily available in public sequence databases. Despite their popularity and utility, we continue to have a poor understanding of polymorphism and copy number variation in rRNA loci across diverse eukaryotic taxa. Quantifying this variation, and pinpointing the selective forces that impact rRNA variation, will be paramount for building a global view of biodiversity, population-level processes, and speciation.

### Number of copies

Although ribosomal copy number variation is known to vary greatly from species to species [7,8], the evolutionary forces responsible for generating such variation have not yet been definitively identified. Eukaryotic cells can possess tens [9] to hundreds [1,10] to tens of thousands [11] of rRNA gene copies across repeated arrays in the nucleus, with copy number exhibiting a strong correlation with genome size regardless of taxonomic group [12]. Only a fraction of the rRNA gene copies are transcribed at any time [13,14], suggesting that the intragenomic variation is not necessarily reflective of cellular requirements. One clear example is *Drosophila*: although species typically possess 200–250 rRNA array copies, deletion studies of rRNA loci indicate that only 35–60 of these units are needed to maintain normal viability in a laboratory setting [15]. In addition, because larger genome size is not necessarily correlated with an increase in protein-coding genes [16], many rRNA copies can remain transcriptionally inactive even during peak periods of organismal growth [13]. Studies in *Daphnia obtusa* [10] and *Drosophila melanogaster* [17] provide further evidence that the rRNA locus is dynamically evolving, and the number of rRNA repeats can expand and contract over short evolutionary times. The excess rRNA copies have been shown to reduce fitness, although the magnitude of the selective disadvantage is currently under debate [5,18]. Regardless, deletions within rRNA loci have been shown to affect expression patterns across thousands of genes, suggesting an important role in determining fitness (and maintaining a spectrum of variation) in natural populations [19].

There are a number of proposed reasons why archaeal and bacterial taxa (unlike eukaryotes) do not show a correlation between genome size and number of rRNA copies [20]. First, copy number variation in eukaryotes may be correlated to eukaryote-specific aspects of recombination or gene repair [6]. Second, copy number variation in bacteria may be limited by nutrient requirements, such that higher copy numbers of rRNA will be favored within more variable and nutrient-rich environments [21]. Third, based on the inverse scaling of effective population size and genome size [22], copy number variation may represent a genomic signal of selection, whereby the relaxed efficiency of selection that accompanies reduced effective population size in eukaryotes may be unable to constrain the accumulation of slightly deleterious non-functional rRNA units. 

### Variation across copies

Within a species, natural selection and concerted evolution typically drive the dominance of one specific rRNA gene variant within individual genomes in a population. The biased process of gene conversion is the primary hypothesized force behind rapid concerted evolution in rRNA, aided by mechanisms such as chromosomal and sister chromatid exchanges [1]. However, given the substantial variation that can exist within genomic rRNA copies [23–25], eukaryotic taxa must be able to maintain some level of rRNA variation that effectively falls under the radar of selection. For example, *Drosophila* species possess 3 to 18 rRNA variants that occur in >3% of genomic ribosomal loci [4]. In *Drosophila* species, intragenomic polymorphisms occur in both 18S and 28S genes with 10-20× higher variation present in noncoding regions of the ribosomal repeats. Purifying selection thus acts within *Drosophila* species to prevent rare rRNA variants from expanding above 5% of the total repeats present within a genome. Similarly, high levels of intragenomic rRNA variation have now been recorded in plant pathogenic fungi [26], despite initial evidence suggesting low levels of sequence diversity in fungal species (where previous methodology was unlikely to capture low level signals from rRNA variants; [9]. Specific ratios of intragenomic rRNA variants were further observed to change over time in *Daphnia* [10], with proportional gene abundances shifting up to 33% between time points.

In *Drosophila*, clusters of rRNA repeats exist as a pair of functional but redundant loci on the X and Y chromosomes [27,28]. To date, there is no evidence that *Drosophila* loci are diverging from one another [1]. However, insights from other eukaryotes such as Planaria [24], aphids [29], and grasshoppers [23] suggest that genomic ribosomal repeats are able to separate into distinct groups or subtypes, although it is not known how commonly this phenomenon may occur across species. For example, fungal species within the Glomeromycota appear to maintain two structurally distinct rRNA variants (L and S), enabled by the physical separation of these loci within the nucleus [30]. The potential existence of multiple, divergent consensus sequences per species has critical implications for sequence-based approaches to biodiversity [31], as it may greatly complicate our ability to derive accurate biodiversity estimates if species are delimited according to the relative abundances of unique rRNA sequences [5]. In addition to multiple genomic rRNA loci, life history traits such as high rates of sexual recombination may encourage the persistence of multiple, and abundant, rRNA variants within a species [9].

In the present study, we aimed to quantify and understand the forces contributing to both intra- and interspecific variation across ribosomal repeat arrays in nematodes. To this extent, we used whole genome shotgun (WGS) data to analyze rRNA repeats in six different species of *Caenorhabditis*. We aimed to assess whether a valid estimate of rRNA diversity could be applied across this diverse phylum, and investigate whether we are able to predict variation in ribosomal array features across different taxa. We also analyzed the rRNA repeats that arose in a *Caenorhabditis elegans* mutation accumulation experiment in order to test the role of selection in determining rRNA repeat copy number. Although little work has been done on rRNA copy number in nematodes, the genome sizes within this phylum (50-250 Mb [32]; fall within the range of most *Drosophila* species (130-364 Mb [33]; and thus we expected our target nematode species to exhibit similar levels of rRNA copy number.

## Materials and Methods

Whole-Genome Shotgun (WGS) sequence reads were obtained from GenBank for the nematode species *Caenorhabditis brenneri, C. remanei, C. briggsae, C. japonica, Brugia malayi*, and *Pristionchus pacificus*. Sequences were subsequently filtered and quality trimmed using LUCY [34]. The ribosomal reference sequence of each species was generated by assembling the WGS sequences in AMOS (http://amos.sourceforge.net/) against the 18S, 5.8S, and 28S gene sequences of *C. elegans* (genome assembly release WS185). Reference bases were determined by the most frequent base call at each position. To ensure the capture of all ribosomal variants, all reads from WGS assemblies were aligned to the reference genome at two levels of maximal divergence (95% and 85%). Remaining gaps in the ribosomal reference were filled in using a combination of low stringency BLAST [35] and MUSCLE [36]. Total WGS coverage was determined by the coverage for all rRNA repeat reads divided by the genome-wide coverage at single copy loci. Since the level of coverage likely varied across rRNA repeat units, this calculation represents an average across the entire repeat array.

WGS sequence reads of each species were aligned against their respective ribosomal reference sequence using AMOS and the base calls at each position were documented. The total copy number of rRNA repeats was determined by dividing the total number of bases aligned by the estimated sequencing coverage depth (total sequenced bases/estimated genome size) for each of the WGS projects. In a similar analysis we utilized 454 and Illumina shotgun reads from *C. elegans* mutation accumulation (MA) lines [37] to evaluate whether the reduced efficiency of selection impacted rRNA copy number. MA line reads were also aligned to the *C. elegans* reference genome sequence and analyzed using identical parameters and approaches as previously described. To assess the uniformity of coverage across the rRNA locus, we aligned the reads from *C. elegans* natural isolate CB4856 (Short Read Archive Project SRR101159 – [38]) , MA41, MA83, and MA99 [37] against the *C. elegans* reference repeat (GenBank accession number X03680.1) using the Burrows-Wheeler Aligner [39].

 To assess error rates in WGS datasets, the observed polymorphic positions in rRNA repeats were compared to the expected distribution of true polymorphisms based on coverage (e.g. for *n*X coverage, an average of *n* reads should be observed for every true polymorphism). To account for sequencing errors, we required a minimum of 50% of 1X coverage depth to consider the base a polymorphism (with 10X coverage, we required at least five identical base calls at that position). This approach was chosen in order to differentiate polymorphic positions from sequencing error. The average sequencing error rate of 454 technology is ~4% [40], while the error rate of Illumina platforms is even lower at ~1% [41]; thus our approach well exceeds the levels of known sequencing errors for high-throughput sequencing platforms.

To further assess how selection impacts rRNA cluster copy number, we performed additional analyses on two natural *C. elegans* isolate strains CB4856 [38] and CB4858 [42]. These were compared to data from MA lines to provide a quantitative measure for the strength of selection in stabilizing rRNA copy number. Following Denver et al. 2005 [43], at neutrality in diploid organisms, we expect the ratio of standing genetic variation (V_g_) to the standing mutational variation (V_m_) in rRNA repeats to be equal to the parameter 4N_e_, where N_e_ is the effective population size. Furthermore, if the amount of standing variation is significantly lower than that expected from mutational variation (V_g_/V_m_ << 4N_e_), we assume that purifying selection is operating on this locus. We calculated V_g_ in rRNA repeats of CB4856 to be 0.0009 (SEM = 3.9x10^-5^) per site and V_m_ in MA41, MA83, and MA99 to be 2.99x10^-5^ (SEM = 1.11x10^-6^), 1.36x10^-5^ (SEM = 7.36x10^-7^), and 1.82x10^-5^ (SEM = 5.90x10^-7^) per site per generation respectively. The joint average of V_m_ across the three MA lines is 2.06x10^-5^ per site per generation, which yields a V_g_/V_m_ of 43.69, and a N_e_ to 10.9.

## Results

The availability of several complete WGS datasets across nematode taxa allowed for an accurate accounting of both copy number and ribosomal sequence diversity. In the natural *C. elegans* isolate (negative control) and three MA lines, we find that the average coverage for 18S (positions 2694-3157), 5.8S (positions 3311-3694), and 26S (positions 3695-7203) are within one standard deviation of the average coverage across the entire repeat. Although the ability to detect extremely small sub-segment expansions may exceed the resolution of this analysis, this data suggests that rRNA repeat expansions and deletions generally involve the entire rRNA cluster. Analysis of six nematode species (Table 1) did not recover any indication of multiple dominant rRNA copies within a genome, supporting a scenario of concerted evolution favoring one dominant rRNA variant that is highly abundant amongst many fewer, low abundance variants. 

**Table 1:** Genomic rRNA copy number estimated from whole genome shotgun data in six nematode species.

Species	*C. brenneri*	*C. remanei*	*C. briggsae*	*C. japonica*	*B. malayi*	*P. pacificus*
Genome Size (Mb)	~150	~135	104	~135	90	169
Gene Count	unknown	~26,000	19,500	unknown	18,500	23,500
Total Bases	21,261,492	11,679,749	3,853,734	4,943,716	9,773,484	11,396,247
Repeat Length	6,929	6,921	6,830	6,825	7,330	6,261
Mean Coverage	3,068	1,688	564	724	1,333	1,820
Coverage Depth	9.5	9.2	10	6.3	8.9	8.9
**Repeat Estimate**	**323**	**183**	**56**	**115**	**150**	**205**

**Figure 1:**
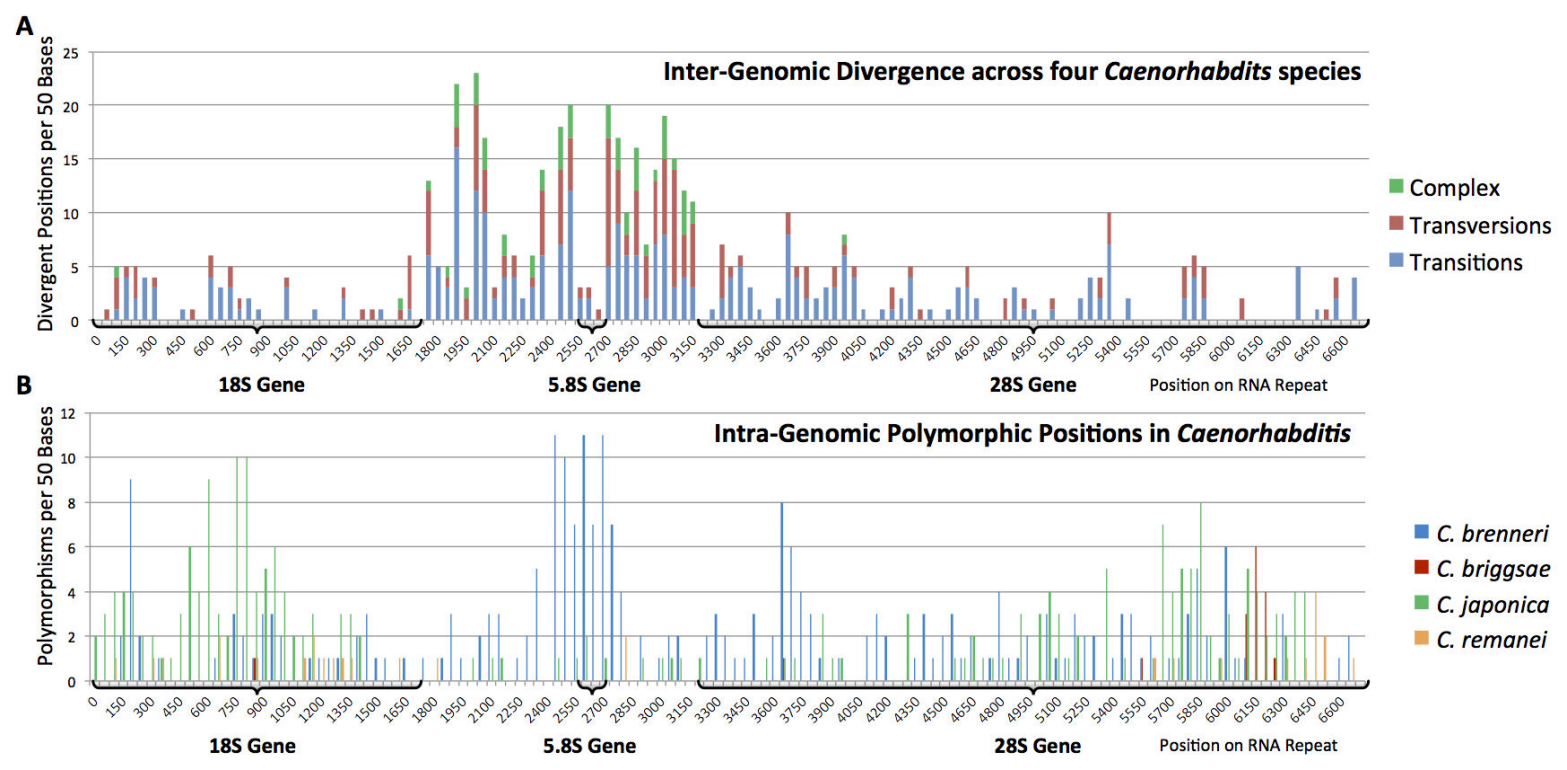
Variation observed in nematode ribosomal arrays. (A) Divergence in rRNA repeats observed between the genomes of *C. elegans*, *C. briggsae*, *C. japonica*, and *C. remanei*; here, base substitutions are denoted as transitions or transversions, while complex polymorphisms represent any type of insertion, deletion, or inversion event. (B) Polymorphic positions in rRNA repeats observed within the genomes of each *Caenorhabditis* species. Results suggest that the pattern of intragenomic polymorphisms is unique across repeats within a species, whereas patterns of interspecific divergence reflect a strong signature of natural selection for rRNA function.

Neither copy number or level of polymorphisms appeared to show any correlation with gene count or genome size in nematodes (Table 1); however, our limited analysis may have precluded the identification of significant correlations such as those previously identified in large datasets [12]. The estimated number of rRNA gene copies varied substantially across taxa, showing a >6 fold difference in repeat copy number across nematode genomes (Table 1). Within a species, the number of complete rRNA repeats appeared to vary widely, with estimates ranging from 56 copies in *C. briggsae* up to 323 copies in *C. brenneri*. Within a nematode genome, higher levels of polymorphism were observed within coding regions of the 18S, 5.8S, and 28S genes as opposed to transcribed but noncoding ITS regions (Figure 1B). The levels of observed polymorphism varied substantially from species to species, and the distribution of polymorphic sites along the ribosomal array showed no overarching patterns across species. Both *C. briggsae* and *C. remanei* exhibited lower levels of polymorphism in rRNA repeats, with fewer numbers of polymorphic positions observed along the entire length of the array. Conversely, *C. brenneri* and *C. japonica* showed extreme variation within rRNA arrays, with some gene positions (18S, 5.8S) displaying >20% polymorphic positions across a 50bp sliding window. Our observations suggest that these patterns of rRNA variation are unique, and potentially genome-specific. However, our approach was unable to determine whether such patterns represent signatures of selection or random stochastic variation. 

In a similar analysis of artificially evolved *C. elegans* mutation accumulation (MA) lines [37], genome data further enabled a robust test for evidence of selection acting on rRNA repeat arrays. MA lines are repeatedly bottlenecked, consistently reducing the effective population size and allowing all but the most deleterious mutations to accumulate. In MA lines, ribosomal copy number exhibited a >2 fold increase in just ~400 generations, with rates of copy number increase calculated at 0.0284—0.3414 rRNA copies per generation (Table 2). These patterns suggest that rRNA copy number is typically constrained by selective pressures in natural populations. To provide further evidence, we also compared the ratio of standing genetic variation in rRNA copy number to the mutational variation (Vg/Vm). Using direct estimates of mutation rate (u) derived from MA experiments [37,44], and silent-site diversity from population data [45], we have previously estimated N_e_ in *C. elegans* to be on the order of 10^6^ [45]. Given that the estimated N_e_ from V_g_/V_m_ in rRNA repeats is 5 orders of magnitude lower than the null expectation, if we assume that *C. elegans* isolate CB4856 is at mutation-selection-drift equilibrium, our data supports the idea that heavy purifying selection is maintaining rRNA copy number in *C. elegans* natural isolates.

**Table 2:** Ribosomal repeat copy number estimates in *Caenorhabditis elegans* displayed for N2 progenitor line and each subsequent MA line.

**Line**	**N2**	41*	83*	99*	**523**	**526**	**529**	**538**	**545**	**553**	**574**
MA Generations	N/A	400	373	420	250	250	250	250	250	250	250
Technology	Illumina	454	454	454	Illumina	Illumina	Illumina	Illumina	Illumina	Illumina	Illumina
Coverage Depth	11.50	1.9	3.1	2.8	9.55	7.38	12.41	12.38	7.01	10.08	17.47
rDNA Coverage	1,295	392	387	716	1,265	1,224	1,727	1,866	839	1,228	2,532
**Repeat Estimate**	**112.6**	**206**	**125**	**256**	**132.4**	**165.8**	**139.2**	**150.7**	**119.7**	**121.8**	**144.9**
**Increase in rRNA repeats** (**unit/generation**)	N/A	**0.2335**	**0.0332**	**0.3414**	**0.0792**	**0.2128**	**0.1064**	**0.1524**	**0.0284**	**0.0368**	**0.1292**

Divergence of ribosomal RNA consensus sequences between four different species showed clear evidence of selection for coding function within conserved gene regions (fewer substitutions), while noncoding regions (ITS) and expansion segments of ribosomal RNA genes (e.g. D2/D3 in 28S) exhibited a higher proportion of transversions and complex polymorphisms (indels and inversions; Figure 1A). These patterns indicate strong purifying selection acting on ribosomal subunit genes, since conservation of ribosome function is critical for maintaining normal cellular processes.

## Discussion

### Implications of rRNA copy number variation

Regardless of the driving mechanisms for intragenomic rRNA polymorphisms and copy number variation, the existence of minor variant gene copies presents substantial challenges for biodiversity estimates and the analysis of marker based datasets [5,31]. High-throughput sequencing technology is able to generate sequence reads representing individual PCR amplicons, allowing a survey of the breadth of variation maintained within a species. On the other hand, Sanger sequencing of PCR products can summarize information from a large pool of PCR products, wherein the signal from the most abundant genomic rRNA copy will generate a unique species-specific “barcode” and allow variant gene copies to remain largely undetectable. At present, it is computationally impossible to distinguish valid, low-level biological variation in eukaryotes (variant rRNA genes) from PCR/sequencing error or taxa representing the “rare biosphere” in high-throughput sequencing datasets [46]. 

For marker gene studies, there is a practical need to quantify and distinguish intragenomic ribosomal diversity from true interspecific signatures. A broad survey incorporating divergent lineages is urgently needed to deepen our understanding of intragenomic ribosomal variation across the Tree of Life. These data will significantly expand our existing knowledge base beyond culturable organisms, and provide a basis for formulating useful diversity metrics enabling interpretation of intragenomic variation in environmental datasets. Quantification of rRNA copy number across diverse genomes could facilitate the application of normalization factors to estimate abundances from environmental data: whereby species X is represented by Y sequence reads, and its genome contains an average of Z rRNA arrays. This methodology would be similar to current approaches in bacterial/archaeal marker gene studies [20], although additional factors such as cell number would need to be considered in multicellular eukaryotes.

The approach used in this study was not limited by comparative alignments, and thus, our results provide a significant advance in the understanding of copy number and polymorphism in the rRNA repeats of diverse nematode species. Here, we are able to show that alignment of WGS sequence to an individual consensus unit of the rRNA array can capture the spectrum of intragenomic variation within an organism. Given that rRNA copy number and polymorphism will directly affect the interpretation of high-throughput sequencing datasets (454/Illumina marker gene studies using environmental rRNA amplicons), methods in the present study were designed to be as inclusive as possible. The present analysis encompasses a number of diagnostic rRNA loci typically applied in environmental studies of eukaryotic diversity (18S [47,48],; D2/D3, [49]. We find that WGS datasets provide critical insights for the interpretation of environmental datasets, and given the observed scale and distribution of polymorphism in ribosomal arrays, it appears that intragenomic variation will continue to present significant problems for the analysis and interpretation of rRNA marker gene data, regardless of diagnostic locus chosen. 

### Polymorphism across nematode rRNA repeats

In nematodes, genomic patterns of polymorphism in the ribosomal gene array displayed a striking similarity to patterns observed in yeast genomes [50], indicating that functional conservation in gene regions and higher, more complex substitution patterns in noncoding regions (Figure 1A) are common across eukaryotic genomes. Although these strong patterns of polymorphism were apparent in each *Caenorhabditis* species, the biological explanation for these observations is presently unclear. Previous studies in fungi [9] have indicated a lack of correlation between rRNA diversity and functional constraint, suggesting that intragenomic diversity across repeats is not subject to selective pressure. Similarly, there appears to be no correlation between ribosomal polymorphism and rRNA copy number in yeast [51]. Polymorphic positions could potentially arise through genomic forces [efficiency of recombination mechanisms, physical separation of rRNA loci, likelihood of orphan events; 6]), or alternatively represent patterns that are a consequence of population-level processes and reproductive dynamics. In yeast, the level of intra-strain polymorphism is correlated with genome structure, whereby SNP patterns indicate a strong differentiation between structured and mosaic genome organization [50]. Therefore, ribosomal polymorphism may have the potential to confer population-level information—and in the case of yeast, allow for separation of “pure” and hybridized strains.

Secondary structure is critical for the integrity and proper functioning of rRNA genes, and even structural modifications within ITS regions can result in nonfunctional rRNAs [5], so it seems unlikely that genomic polymorphisms reflect the accumulation of rRNA pseudogenes. The accumulation of nonfunctional rRNA copies is expensive in terms of fitness, although some pseudogenes can coexist alongside functional variant rRNA gene copies [5]. In fungi, variant gene copies that persist within populations can even exhibit severe reductions in structural complexity without loss of function [30]. In addition, nonfunctional gene copies are less likely to accumulate in small, compact nematode genomes [52,53], and it is well known that the similar-sized *Drosophila* genome is characterized by a relative lack of pseudogenes [54]. 

Transposable element insertions, multiple chromosomal loci for rRNA arrays, or epigenetic influences (methylation or silencing effects) are all mechanisms thought to limit concerted evolution within a species. Evidence from *Daphnia* [55] suggests that transposons inserted within rDNA loci can inhibit homogenization across arrays, effectively maintaining cryptic genetic variation in ribosomal RNA genes and even prolonging the lifespan of deleterious mutations (rRNA variants with lower thermal stability). Such novel transposition mechanisms may also drive differences in chromosomal arrangement and number of genomic rRNA loci within a species [56]. Evidence of methylation has been reported in the grasshopper *Podisma pedestris* [23], a species where ineffective concerted evolution has maintained divergent groups of genomic rRNA loci. Previous evidence has suggested that the silencing of rRNA genes (e.g. through methylation and heterochromatization) can significantly reduce the homogenizing forces of concerted evolution [57]. In addition, reductions in rRNA copy number are accompanied by a release of silencing factors such as Sir2 [58],, producing an increase in telomere silencing. Given that rRNA genes are highly transcribed and tightly controlled, any genomic modifications at rRNA loci (changes in gene copy number, differential upregulation/suppression of rRNA variants) will translate into “extra-coding functions” impacting a multitude of cellular processes [6].

It is thought that selection will act above a threshold level of divergence present within an individual ribosomal unit, suggesting that a common level of diversity (falling under the radar of selection) can exist within the genome of any given species. Indeed, such low-level rRNA variation appears to be typical across yeast strains isolated from disparate geographic locations [50]; 80% of polymorphic positions in gene regions (18S/26S) were reported to have frequencies <10% across genomic rRNA arrays. Although members of a population tend to share similar frequencies of intragenomic rRNA variants, rare individuals may display markedly divergent proportional abundances of ribosomal gene variants [59]. Maintaining a diverse arsenal of variant rRNA copies (cryptic gene variation) has thus been implicated as a type of pre-adaptation to new environments [60] or host-associated habitats [25]. In free-living eukaryotes, higher levels of intragenomic rRNA variation may confer subtle advantages for opportunistic taxa that must physiologically adapt to varying environmental conditions (e.g. through long-distance dispersal). 

### Copy number and selection

In the present study, the reduced efficiency of selection in *C.elegans* MA lines resulted in a consistent expansion of genomic rRNA copy number. While parent N2 strains maintained ~112 rRNA repeats, the estimated copy number increased to 119-256 repeats in ten independent lines subjected to repeated bottlenecking for >250 generations (Table 2). Significant changes in rRNA copy number have also been observed in asexually propagated *Daphnia obtusa* lines (bottlenecked strains exhibiting 53-233 repeats, from an estimated ~160 copies in the stem mother [10]). Copy number expansion is not exclusive to nematodes, and evidence for copy reduction in *Daphina* suggests that the removal of selective pressures can elicit differential responses in rRNA loci. Insight from bottlenecked MA lines, and our quantitative analysis of mutational variation (V_g_/V_m_) in natural *C. elegans* isolates, strongly suggests that rRNA copy number may typically be constrained by selective forces in natural populations. The dynamic nature (and thus instability) of the rDNA locus makes it a particularly fragile site within the genome—while DNA repair mechanisms may function to reduce rRNA copy number through recombination-mediated loss, gene amplification during replication can also effectively increase the number of genomic rRNA repeats [6]. Such gain or loss may occur as frequently as once per cell division [61]. Alternatively, rRNA expansion may occur via independent mutations unrelated to mechanisms for homologous recombination; reproducible expansion of rRNA repeats was observed in yeast cells lacking the histone chaperone protein Asf1 [62]. Thus, natural selection may lend favor to a narrow range of repeat copy number, dictated by a mutation-selection balance. Within a species, higher or lower overall copy number could perhaps be determined by sequence-specific features in dominant rRNA variants (e.g. a threshold needed to maintain genome stability). 

The tendency for rRNA copy number to expand in *C. elegans* MA lines suggests a non-random pattern and specific rules potentially governing this phenomenon. On a large scale, genomic rRNA copy number may correlate with life history traits and effective population size (N_e_). Species with very small N_*e*_ should exhibit higher rRNA copy number, as ribosomal loci expand under the reduced efficiency of selection (assuming no fitness cost is incurred for higher copy number). In contrast, purifying selection may strictly limit the maximum number of genomic rRNA loci in taxa with a large N_*e*_ since higher copy numbers may confer a fitness disadvantage amongst many conspecific individuals. Higher rRNA copy number has been linked with faster growth rates [63,64], but loss of rRNA copies appears to be a frequent genomic event [61]; promoter sites such as E-Pro [65] likely govern the recovery and expansion of rDNA loci once copy number falls below a minimum threshold and begins to impair fitness. 

A growing body of evidence is now emphasizing the link between genomic rRNA patterns and ecology [21]. Higher copy number, and putatively higher expression of ribosomal RNA genes, requires a substantial source of phosphorus—oftentimes a limited nutrient in habitats with reduced food input or low food quality. Elser et al. [66] have hypothesized that species assemblages and community interactions, as well as biogeochemical nutrient cycling, are largely driven by genomic variation across rRNA loci in different taxa. Under their proposed scenario, higher rRNA copy number should allow for fast growth and rapid exploitation of available ecological niches—however, this strategy requires a significant amount of phosphorus to satisfy cellular ribosomal transcription, and such taxa may become locally extinct when high-quality food sources become exhausted. In contrast, slower growing taxa (with lower rRNA copy number and less phosphorus requirements) can persist through adverse environmental conditions and tolerate even the lowest quality food. The plasticity of genomic rRNA loci may be further influenced by trophic interactions, where genomic patterns are shaped though environmental parameters and ecological forces. 

Ultimately, copy number and polymorphism in ribosomal RNA genes may be determined by a combination of genomic forces (mutation, drift, and selection) and environmental conditions (nutrient availability, competition). Although there is a substantial body of literature on rRNA gene arrays, our understanding of intra- and interspecific variation in these loci remains rudimentary at best. The present study provides further insight into genomic patterns and possible mechanisms impacting the evolution of ribosomal arrays in nematodes. Future investigations must expand this focus across diverse eukaryote species, in order to inform and complement environmental biodiversity studies relying on high-throughput sequencing approaches.

### Data Access

Nematode WGS trace datasets (*Caenorhabditis*, *C. remanei, C. briggsae, C. japonica, Brugia malayi*, and *Pristionchus pacificus*) are public datasets accessible via NCBI’s ftp server (ftp://ftp.ncbi.nih.gov/pub/TraceDB/). All other data is accessible within the NCBI Short Read Archive (*C. elegans* N2 and MA lines accession no. SRA009375, data from [36]; *C. elegans* natural isolates Bioproject accession numbers CB4856, data from [37], and CB4858, data from [41]). 
